# Survival impact of the time gap between surgery and chemo-radiotherapy in Glioblastoma patients

**DOI:** 10.1038/s41598-020-66608-3

**Published:** 2020-06-12

**Authors:** Inbar Zur, Tzahala Tzuk-Shina, Marina Guriel, Ayelet Eran, Orit Kaidar-Person

**Affiliations:** 1 Oncology Institute, Rambam Medical Center, Haifa, Israel; 2 Neuro-oncology unit, Rambam Medical Center, Haifa, Israel; 3 Neuro-Radiology unit, Rambam Medical Center, Haifa, Israel; 4 Radiation Oncology unit, Rambam Medical Center, Haifa, Israel; 5 Radiation Oncology, Sheba Tel Hashomer, Ramat Gan, Israel

**Keywords:** CNS cancer, Neurology

## Abstract

Glioblastoma treatment protocol includes chemo-radiation (CRT) after maximal safe resection. However, the recommended time-gap between surgery and CRT is unclear, most trials protocol required an interval of less than 6 weeks. In the current study we evaluated the association of the time-gap between surgery and CRT to overall survival (OS) and progression free survival (PFS) in a tertiary center. After ethics committee approval, a retrospective study was conducted. Data was collected from the medical records of consecutive glioblastoma patients treated between 2005–2014. Parameters of interest included: background characteristics of patients, treatment dates and type of treatment, treatment interruptions and survival. Only patients who were diagnosed with WHO IV, underwent surgical resection (any type), and treated with postoperative CRT were included. For the analysis, patients were divided into 3 groups according to the time gap from surgery to CRT: <4 weeks, 4–6 weeks and >6 weeks. Overall survival and PFS were investigated using the Kaplan-Meier method and Cox proportional hazard model. Out of 465 patients, 204 were included. Median age was 60 years (range: 23–79 years) and 61.7% male vs. 38.3% female. There was a significant difference in OS (HR = 0.49, p-value = 0.002, 95% CI: 0.32–0.78) and PFS (HR = 0.51, p-value = 0.003, 95% CI: 0.33–0.79) in the group who was treated with CRT 6 weeks or more after surgery, compared with the other two groups tested. In our study, 6 weeks or more time-gap (median of 8 weeks) between surgery and CRT was associated with better OS and PFS among newly diagnosed glioblastoma patients. Our results are probably subjected to unaccounted biases of a retrospective study, and that CRT in this patient population is an effective therapy that overcomes the potential harm of initiating therapy later than 6 weeks. Our current approach is to initiate CRT within 6 weeks after surgery, similar to what is recommended in the literature, but the data from this study provide us with information that no major harms was done in patients who were delayed.

## Introduction

Glioblastoma is the most common primary CNS malignancy in adults, with incidence of 2–3 new cases in 100,000 people per year among most of Europe and North America countries^1^. As a tertiary hospital, that serves the population in the North of Israel, we treat approximately 50 new glioblastoma patients every year.

Glioblastoma remains an aggressive tumor, untreated patients usually have OS of only a few months, and even though the standard treatment in patients who have good performance status is a three-modality approach of maximal safe resection, postoperative chemo-radiation (CRT) with Temozolomide (TMZ) and adjuvant TMZ^2^, the 2-year OS still remains as low as about 25%^1^. Patients who are at good performance status, undergo gross surgical resection and standard postoperative therapy can survive up to 14.6 months^3^. This protocol was published by Stupp *et al*., in 2005^4^ and since then many trials failed to show significant survival advantage^5,6^. In 2015 a phase III randomized trial evaluating the addition of Tumor-Treating-Fields (TTFields) to maintenance of TMZ chemotherapy vs maintenance TMZ alone, resulted in statistically significant improvement in progression-free survival and OS^7^.

Importantly in the randomized trials evaluating the treatment in glioblastoma, the radiation therapy (RT) (with TMZ) is given no later than 6 weeks after surgery. As a tertiary referral center, RT is often delayed more than 6 weeks. Therefore, the aim of our study was to evaluate the time-gap from surgery to CRT in glioblastoma patients at our hospital and to evaluate its impact on disease outcome.

## Methods

The study was approved by the Institutional (Rambam Medical Center) Review Board Approval (IRB), and patient’s consent was waived by the IRB due to the retrospective nature of the study. All methods were performed in accordance with the relevant guidelines and regulations. We conducted a retrospective study of all medical records of patients who were diagnosed with high grade glioma treated at our institution between 2005–2014.

Only patients who underwent surgical resection (any type) for primary high grade glioma, who had histologic confirmation of glioblastoma (WHO IV only) and were planned for CRT (TMZ with 60 Gy in 30 fractions, and adjuvant TMZ) were included in the final analysis.

Data recorded from the medical records included patient- and tumor- related characteristics such as: age, gender, comorbidities, steroid use, time at first imaging testing, date of surgical resection, extent of surgery (biopsy, sub-total resection or gross total resection), first neuro-oncological evaluation, time of first RT planning, first day of RT, last day of RT, dose and fractionation, RT treatment interruptions, first day of adjuvant chemotherapy (TMZ), last day of TMZ treatment, number of adjuvant TMZ cycles, type and timing of second line systemic therapy, and re-surgery.

### Statistical analyses

T-test was used for continuous variables in order to test the variability between the group of patients treated within 6 weeks from diagnosis (surgery) and the group of patients who were treated after 6 weeks following diagnosis.

Univariate analyses were carried out using Pearson -chi-square or Fisher’s exact test for comparing categorical variables. A probability value < 0.05 was considered statistically significant.

The time gap between surgery and initiation of CRT was analyzed as a continuous variable and then again as a categorical variable based on 3-time intervals, as follows: <4 weeks, 4–6 weeks and >6 weeks. Overall survival was defined as time between surgery and time of death. To compare baseline characteristics among the different groups, contingency tables were generated using Pearson’s chi square test for categorical variables, and Kruskal-Wallis test for continuous variables. Univariate analyzes (Kaplan-Meier) was made in order to test survival rates in categorical variables: extent of surgery, RT interruption, steroidal treatment and gender. In multivariate analyzes (Cox proportional hazard model), a model was adjusted using the following variables, which were found to be statistically significant in univariate analyzes: age, extent of surgery, co-morbidities, total RT dose (Gy), number of adjuvant TMZ cycles and switching to 2nd line treatment. Additionally, the end goal - variable of time interval from surgery until combined therapy was also included. Progression free survival (PFS) was defined as the time of tumor progression documented by imaging or clinical deterioration following completion of the first combined treatment with RT + TMZ.

## Results

A total of 465 patients were diagnosed and treated at our center for high grade glioma during 2005–2014. Out of these patients, 204 (43.87%) patients were included in the final statistical analysis. Median age was 60 years (range: 23–79 years), 126 patients were in the age group ranging between 40–64 years old (61.7%). and male to female ratio was 1.6 (61.7% male vs. 38.3% female)

All patients underwent a surgical procedure: 22.77% underwent biopsy only (n = 46), 63.37% underwent gross total resection (GTR) (n = 128) and 13.86% underwent sub-total resection (STR) (n = 28).

Following surgery, all patients were treated with combined CRT according to the following time-gaps: 47 patients (23.04%) began CRT within 4 weeks after surgery; 72 patients (35.29%) began therapy between 4 to 6 weeks after surgery; and the remaining 84 patients (41.18%) began CRT more than 6 weeks after surgery (median 56 days, range 43–154 days).

Most patients (88.3%, n = 166 (completed a total RT dose of >56 Gy. Patients’ characteristics and treatment information according to the time-gap groups is presented at Table 1.

**Table 1:** Patients’ and treatment characteristics according to the time-gap to CRT.

Variant	Total, n (%)	<28 d (%)	35–42 d (%)	> 42 d (%)	p - value
Age at diagnosis, n	204	47	72	84	0.39
<40 y	11 (5.4)	3 (6.4)	5 (7)	3 (4)	
40–64 y	126 (61.7)	24 (51)	46 (64)	56 (64	
>64 y	67 (32.8)	20 (42.6)	21 (29)	25 (32)	
Gender, n	204	47	72	84	0.5
Male	126 (61.7)	28 (59.6)	42 (59)	56 (67)	
Female	78 (38)	19 (40. 4)	30 (41)	28 (33)	
Extent of surgical resection, n	202	45	72	46	0.059
Gross total resection	128 (63)	21 (46)	46 (64)	23 (50)	
Subtotal resection	28 (13.9)	8 (18)	9 (12)	10 (21)	
Biopsy	46 (22.7)	16 (36)	17 (24)	13 (29)	
MGMT status, n	38	11	7	20	0.17
Positive	16 (42)	4 (36)	1 (14)	11 (55)	
Negative	22 (57.9)	7 (64)	6 (86)	9 (45)	
Comorbidities at diagnosis, n	147	39	52	55	
Diabetes Mellitus	44 (29.9)	11 (28)	16 (31)	17 (31)	0.9
Hypertension	93 (63)	24 (62)	32 (62)	36 (65)	0.65
Ischemic heart disease	10 (6.8)	4 (10)	4 (7)	2 (4)	0.28
RT total dose (Gy), n	188	40	67	80	0.047
≥56	166 (88.3)	31(77.5)	60 (90)	74 (93)	
36–50	17 (9)	7 (17.5)	4 (6)	6 (7)	
≤32	5 (2.6)	2 (5)	3 (4)	0 (0)	
No. of adjuvant TMZ cycles*, n	200	45	70	84	0.88
0	18 (9)	4 (9)	6 (9)	8 (10)	
1–3	80 (40)	22 (48)	23 (33)	35 (42)	
4–6	47 (23.5)	8 (18)	19 (27)	20 (24)	
7–9	19 (9.5)	4 (9)	8 (11)	7 (8)	
≥10	36 (18)	7 (16)	14 (20)	14 (16)	
No. of Patients who switched to 2nd line therapy	204	47	72	84	0.2
Yes	114 (56)	21 (45)	41 (57)	51 (61)	
No	90 (44)	26 (55)	31 (43)	33 (39)	
Pauses during RT	188	38	69	80	0.4
Yes	21 (11)	2 (5)	4 (6)	14 (18)	
No	167 (89)	36 (95)	65 (94)	66 (82)	
Steroid use during RT	201	46	71	83	0.29
Yes	181 (90)	40 (87)	67 (94)	73 (88)	
No	20 (10)	6 (13)	4 (6)	10 (12)	
*Post concomitant therapy with RT + TMZ.					

CRT- chemoradiation; RT- radiation therapy; TMZ-tomozolamide; MGMT – methylguaninemethyltransferase.

P < 0.05 is statistically significant.

Twenty-one patients (11%) had treatment interruptions at time of CRT due to a variety of reasons (hospitalization, allergic reaction to therapy, drop in blood count) and 181 (90%) received steroid treatment while treated with combined therapy.

TMZ adjuvant therapy (after completion of CRT) was as follow: 40% (n = 80) received 1–3 cycles; 23.5% (n = 47) received 4–6 cycles; 9.5% (n = 19) received 7–9 cycles; and 18% (n = 36) received 10 or more cycles of TMZ. Only 9% (n = 18) were not treated with additional cycles of TMZ. None of the patients were treated with Tumor-Treating-Fields. Four patients were lost to follow-up.

Two of those were lost to follow up received 5–6 cycles of adjuvant therapy with TMZ and were included in the final analysis. The other two patients had been preliminary excluded from the study due to discordance with inclusion criteria (i.e. avoidance from RT and lack of sufficient data).

Second line therapy after adjuvant TMZ was given to 114 patients, (55.9%).

### Survival analysis

Tables 2–5 summarizes the univariate and multivariate for PFS and OS. The univariate analysis shows that worse PFS was associated with older age (>64 years), hypertension, ischemic heart disease, subtotal resection (STR), and patients who completed only total dose of 32 Gy or less. These were also found to be associated with worse PFS in the multivariate analysis. A time-gap of >6 weeks was associated with better outcome (HR = 0.51, p-value = 0.003).

**Table 2:** Univariate analysis for progression free survival.

Variant	Hazard ratio	95% CI (Lower)	95% CI (Upper)	p-value
**Age at diagnosis, n**				
<40 y	1			
40–64 y	1.695	0.853	3.368	0.132
**>64 y**	**2.529**	1.232	5.192	**0.011**
**Sex, n**				
Male	1			
Female	0.913	0.668	1.845	0.570
**Extent of surgical resection, n**				
Gross total resection	1			
**Subtotal resection**	**2.197**	1.430	3.375	**0.000**
Biopsy	1.394	0.943	2.060	0.095
**MGMT status, n**				
Positive	0.811	0.398	1.651	0.563
Negative	1			
**Comorbidities at diagnosis, n**				
Diabetes Mellitus	1.269	0.873	1.845	0.211
**Hypertension**	**1.385**	1.016	1.887	**0.039**
**Ischemic heart disease**	**3.108**	1.506	6.412	**0.002**
**RT total dose (Gy), n**				
≥ 56	1			
36–50	1.236	0.697	2.189	0.468
**≤32**	**13.120**	5.175	33.263	**0.000**
No. of adjuvant Temozolomide cycles*, n				
0	1			
1–3	1.290	0.681	2.442	0.434
4–6	1.030	0.525	2.023	0.931
7–9	0.482	0.226	1.027	0.059
≥10	0.297	0.152	0.582	**0.000**
**Patients who switched to 2nd line therapy**				
Yes	0.898	0.656	1.229	0.502
No	1			
**Pauses during RT**				
Yes	0.746	0.443	1.255	0.269
No	1			
**Steroid use during RT**				
Yes	1.485	0.868	2.542	0.149
No	1			
*Post concomitant therapy with RT + TMZ.				

Hazard ratio (HR) less than 1 – better overall survival, >1 – worse overall survival; P-value < 0.05 – statistically significant.

**Table 3:** Multivariate analysis for progression free survival.

Variant	Hazard ratio	95% CI (Lower)	95% CI (Upper)	p-value
**Age at diagnosis, n**				
<40 y	1			
40–64 y	1.701	0.778	3.720	0.184
>64 y	2.139	0.907	5.046	0.082
**Extent of surgical resection, n**				
Gross total resection	1			
**Subtotal resection**	**3.142**	1.944	5.077	**0.000**
Biopsy	1.174	0.754	1.828	0.477
**Comorbidities at diagnosis, n**				
Hypertension	0.882	0.616	1.263	0.494
**Ischemic heart disease**	**1.484**	2.007	9.702	**0.000**
**RT total dose (Gy), n**				
≥56	1			
36–50	0.787	0.365	1.694	0.540
**≤32**	**8.138**	2.760	23.992	**0.000**
No. of adjuvant Temozolomide cycles*, n				
0	1			
1–3	0.734	0.304	1.774	0.493
4–6	0.631	0.258	1.540	0.312
7–9	0.229	0.085	0.612	**0.003**
≥10	0.156	0.064	0.377	**0.000**
**Waiting time until concomitant therapy with RT** + **TMZ, d**				
<28 days	1			
28–42 days	0.834	0.530	1.313	0.433
≥42 days	0.514	0.330	0.799	**0.003**
*Post concomitant therapy with RT + TMZ.				

Hazard ratio (HR) less than 1 – better overall survival, > 1 – worse overall survival; P-value < 0.05 – statistically significant.

**Table 4:** Univariate analysis for overall survival.

Variant	Hazard ratio	95% CI (Lower)	95% CI (Upper)	p-value
**Age at diagnosis, n**				
<40 y	1			
40–64 y	2.162	1.093	4.275	**0.027**
>64 y	3.463	1.708	7.020	**0.001**
**Sex, n**				
Male	1			
Female	0.881	0.660	1.177	0.393
**Extent of surgical resection, n**				
Gross total resection	1			
Subtotal resection	1.334	0.878	2.026	0.177
Biopsy	1.677	1.187	2.370	**0.003**
**MGMT status, n**				
Positive	0.762	0.395	1.471	0.418
Negative	1			
**Comorbidities at diagnosis, n**				
Diabetes Mellitus	1.478	1.055	2.072	**0.023**
Hypertension	1.363	1.028	1.806	**0.031**
Ischemic heart disease	3.493	1.825	6.683	**0.000**
**RT total dose (Gy), n**				
≥56	1			
36–50	1.789	1.081	2.962	**0.024**
≤32	6.556	2.638	16.297	**0.000**
No. of adjuvant Temozolomide cycles*, n				
0	1			
1–3	0.896	0.534	1.506	0.680
4–6	0.694	0.400	1.202	0.193
7–9	0.372	0.193	0.718	**0.003**
≥10	0.180	0.099	0.327	**0.000**
**No. of Patients who switched to 2nd line therapy**				
Yes	0.445	0.334	0.594	**0.000**
No	1			
**Pauses during RT**				
Yes	0.817	0.507	1.317	0.407
No	1			
**Steroid use during RT**				
Yes	1.453	0.880	2.398	0.144
No	1			
*Post concomitant therapy with RT + TMZ.				

Hazard ratio (HR) less than 1 – better overall survival, > 1 – worse overall survival; P-value < 0.05 – statistically significant.

**Table 5:** Multivariate analysis for overall survival.

	Hazard ratio	95% CI (Lower)	95% CI (Upper)	p-value
**Age at diagnosis, n**				
<40 y	1			
40–64 y	1.733	0.788	3.813	0.171
>64 y	2.152	0.914	5.068	0.080
**Extent of surgical resection, n**				
Gross total resection	1			
**Subtotal resection**	**1.917**	1.216	3.024	**0.005**
Biopsy	1.253	0.828	1.897	0.285
**Comorbidities at diagnosis, n**				
Diabetes Mellitus	1.215	0.832	1.774	0.314
Hypertension	0.767	0.550	1.069	0.117
**Ischemic heart disease**	**3.236**	1.553	6.744	**0.002**
**RT total dose (Gy), n**				
≥56	1			
36–50	1.106	0.558	2.193	0.772
**≤32**	**3.336**	1.236	9.000	**0.017**
No. of adjuvant Temozolomide cycles*, n				
0	1			
1–3	1.131	0.515	2.484	0.759
4–6	1.093	0.503	2.374	0.823
7–9	0.541	0.221	1.323	0.178
≥10	0.206	0.092	0.464	**0.000**
**Patients who switched to 2nd line therapy**				
Yes	1			
No	0.494	0.351	0.695	**0.000**
**Waiting time until concomitant therapy with RT** + **TMZ, d**				
<28 days	1			
28–42 days	0.867	0.561	1.339	0.520
≥42 days	0.498	0.319	0.777	**0.002**
*Post concomitant therapy with RT + TMZ.				

Hazard ratio (HR) less than 1 – better overall survival, > 1 – worse overall survival; P-value < 0.05 – statistically significant.

**Figure 1:**
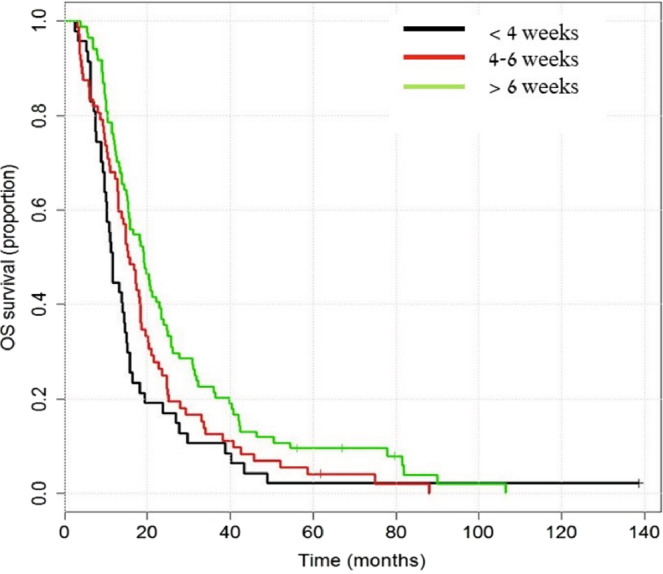
Overall survival by interval between surgery and combined therapy (TMZ + RT). Longrank test for difference in overall survival: p-value = 0.0092.

The results of the univariate analysis of OS are presented in Table 4. Older age, diabetes mellitus, and a total dose of 32 Gy or less, were associated with worst OS outcome. A time gap of more than 6 weeks from surgery to CRT was associated with statistically significant better OS (HR = 0.49, p-value = 0.002). The results of multivariate analysis are presented in Table 5. A Kaplan- Meier method for OS is presented in Fig. 1.

Additional statistical analysis was done for the group of patients who completed a total RT dose of 56–60 Gy (n = 166). The median age was 59 (range: 23–76), 68% were between the age of 40–60 years, 62% of the patients were male. There were no significant differences in the characteristics between the different gap-time groups except that the groups that was treated in a time-gap >6 weeks had significant more treatment interruptions (<4 weeks: 2 out of 28; 4–6 weeks: 2 out of 59; >6 weeks: 12 out of 74), p = 0.042.

**Figure 2:**
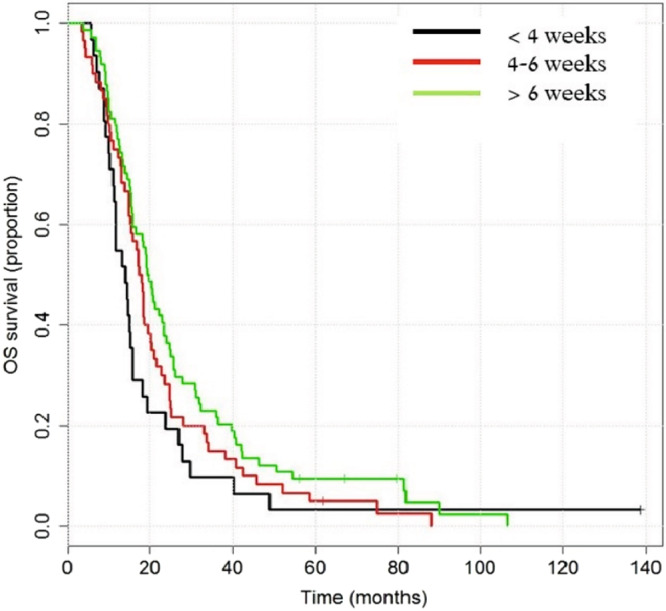
Overall survival in a subgroup of patients who completed a total radiation dose of 56–60 Gy, according to the interval between surgery and combined therapy (TMZ + RT) Longrank test for difference for overall survival: p-value = 0.097.

**Figure 3:**
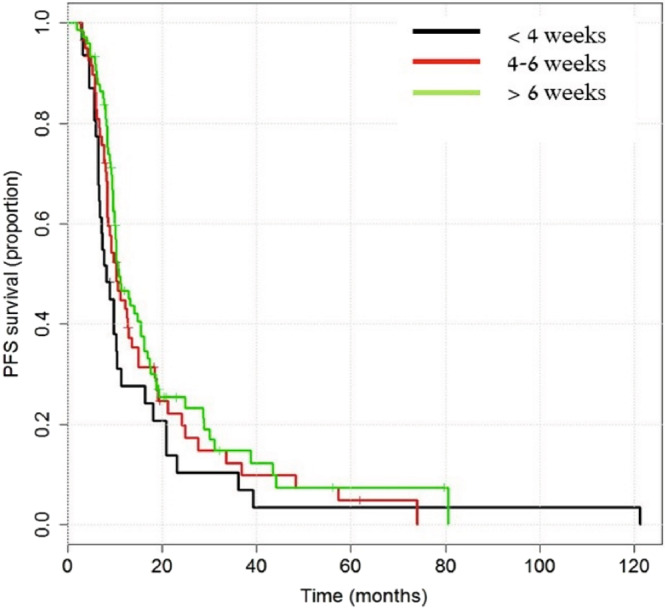
Progression Free Survival in a subgroup of patients who completed a total radiation dose of 56–60 Gy, according to the interval between surgery and combined therapy (TMZ + RT). Longrank test for difference for progression-free survival: p-value = 0.1853.

Multivariate analysis for PFS and OS showed that a time-gap of > 6 weeks from surgery to CRT was associated with better outcome (Table 6, multivariate analysis for OS). However, a Kaplan- Meier method for PSF or OS in this population (Figs. 2, 3), did not show significant differences between the 3 different time intervals that were tested (p-value > 0.05).

**Table 6:** Multivariate analysis for overall survival in patients who received 56–60 Gy.

Variant	Hazard ratio	95% CI (Lower)	95% CI (Upper)	p-value
**Age at diagnosis, n**				
<40 y	1			
40–64 y	1.557	0.714	3.397	0.266
>64 y	1.985	0.856	4.599	0.110
**Extent of surgical resection, n**				
Gross total resection	1			
Subtotal resecction	1.673	1.023	2.739	**0.041**
Biopsy	1.096	0.708	1.698	0.680
**Comorbidities at diagnosis, n**				
Ischemic heart disease	2.838	1.202	6.701	**0.017**
No. of adjuvant Temozolomide cycles*, n				
0	1			
1–3	0.695	0.317	1.522	0.363
4–6	0.653	0.293	1.453	0.296
7–9	0.329	0.134	0.805	**0.015**
≥10	0.123	0.052	0.288	**0.000**
**Patients who switched to 2nd line therapy**				
Yes	1			
No	0.457	0.319	0.654	**0.000**
**Waiting time until concomitant therapy with RT** + **TMZ, d**				
<28 days	1			
28–42 days	0.910	0.572	1.449	0.692
≥42 days	0.568	0.358	0.899	**0.016**
*Post concomitant therapy with RT + TMZ.				

Hazard ratio (HR) less than 1 – better overall survival, > 1 – worse overall survival; P-value < 0.05 – statistically significant.

## Discussion

The aim of the current study is to evaluate the impact of the time-gap between surgery and CRT in patients who were diagnosed with glioblastoma. This study presents real world data of patients treated at a tertiary center. Interestingly, our results demonstrate that glioblastoma patients who had begun combined therapy with RT + TMZ *after* 6 weeks or more had significant better OS and PFS compared to the other two groups of patients who had started the same treatment earlier. According to current treatment recommendation, CRT should be initiated within the first 6 weeks after surgery. A large collective analysis, aimed to evaluate the recommended time-gap between surgery to CRT, combined the data from 16 the Radiation Therapy Oncology Group (RTOG) randomized controlled trials and concluded that PFS is not negatively affected with delay until RT initiation, as long as RT has been initiated *within the first six weeks* after surgery^8^. Moreover, recent study, published in early 2018, concluded that CRT with TMZ should be initiated *within 5–6 weeks* past surgery, as recommended in current treatment guidelines^9^. However, a publication by Wang *et al*., which included 447 glioblastoma patients, found that patients who had undergone RT within 3 weeks or less after surgery tended to have worse prognostic factors (advanced age, low KFS, biopsy alone) compared to patients who had undergone RT after 3 weeks. Additionally, delay of RT after surgery was not shown to significantly affect OS^10^. This conclusion had been supported by other studies^8,11^. Having that said, few studies present the opposite conclusion and claim to find a direct negative prognostic effect with delay in waiting time until RT^2,12,13^. Lawrence *et al*., conducted meta-analysis comparing a number of studies targeting this issue, and concluded that 4–6 week time gap from surgery to RT is safe and offer a moderate advantage, yet there is no justification in waiting time further than six weeks^14^.

Even though there were no significant differences in between the time-gap groups in our initial analysis, it might be that the shorter time gap group was too small to detect these differences, and Table 1 shows that these patients tend to have less favorable characteristics (e.g., older, less GTR). Therefore, it might be that there is an accounted bias between the study groups, and that patients who were “worse” at presentation were treated earlier. Moreover, as a retrospective study, there might be confounding factors that are unaccounted (such as other morbid conditions, neurologic status, tumor mutational status). As opposed to a randomized control trial where the trial population is stratified to allow for a balanced comparison. The total radiation dose is a significant factor for outcome in these patients. Ninety three percent of the patients in the group who were treated at an interval of > 6 weeks received 56–60 Gy, compared to 90% in the group 4–6 weeks and 77.5% in the group <4 weeks. This might indicate that these patients were at better KPS that allow them to complete treatment. When controlling population characteristics and addressing the cohort of patients treated with 56–60 Gy RT in aim to reduce the unaccounted biases, the trend remains the same, the time-gap of > 6 weeks was still associated with better outcome. However, Kaplan- Meier analysis did not demonstrate a significant difference between the 3 time-gap tested.

There are several limitations to our study. Since this is a retrospective study and the performance status was not reported in many of the cases this was not included in the analysis which might be is a major flow as Karnofsky Performance Status (KPS) score is proven to be significant for survival and therefore we did not evaluate the RPA classification of the different study groups^9^. Moreover, tumor mutations in isocitrate dehydrogenase (IDH) 1 and 2 was only available in later years (after 2010) and MGMT is not done in all patients. As MGMT done in a different laboratory and often takes a few weeks, our department protocol is not to send MGMT to all patients if they are planned for CRT, in order not to delay treatment. If treatment with TMZ alone is considered (because of age, poor performance status) than our protocol is to send for MGMT prior to treatment. Therefore, IDH and MGMT data are not available for all patients and for further analysis. Another flaw is that we did not control for the use of steroids. If possible, our policy is to stop or reduce the steroid dose to minimum possible before CRT. However, often patients are re-started on steroids at time of CRT because of side-effects of radiation and edema. Nevertheless, other prognostic factors that were found associated with worse OS and PFS were similar factors reported by others, therefore, our study represent a similar population reported in other studies. Moreover, nowadays, Tumor-Treating-Fields as an adjuvant treatment at time of maintenance TMZ is available in our country for treatment of GBM patients^7^. Our study did not analyze the contribution of second line therapy. It is our policy to discuss all cases at a multidisciplinary meeting which includes neuro-oncologists, neuro-radiologist, radiation oncologist and neurosurgeons. After a progression is decided (imaging, clinical status, etc) we discuss the treatment approach per case. At that time, most cases, we treated with second line bevacizumab. If patients were at good KPS, and location of the lesion is favorable, we do consider re-resection, and if there was a long interval from RT, and no suspected RT-related toxicity, and volume/location of the lesion allows, we consider re-RT with/without bevacizumab. However, none of these approaches were clearly shown to be associated with improvement in survival and each approach is significantly influenced by confounding factors (e.g., late progression of a small lesion and a favorable location versus early progression in a symptomatic patient).

Our study reflects real-world data from a tertiary facility. Our radiation unit is often subjected work overload of the staff (i.e., radiation oncologists, physicists) and no slots on the treatment machines, leading that the time to initiation of RT (therefore CRT) can be prolong compared to less busy centers. We do believe that the unaccounted bias for better/worse prognosis between the groups is a major contributor to the study results (e.g., KPS, IDH, MGMT). The KPS is a key factor that is missing from our data and might explain why the >42 days group had a higher RT dose and more treatment lines. However, this group had more treatment interruption because of side effects (Table 1), nevertheless, CRT in this patient population is an effective therapy that overcomes the potential harm of initiating therapy later than 6 weeks.

Our current approach is to initiate CRT within 6 weeks after surgery, similar to what is recommended in the literature, but the data from this study provide us with information that no major harms was done in patients who were somewhat delayed.
